# Prevalence and associated factors of birth trauma in Ethiopia: A systematic review and meta-analysis

**DOI:** 10.1371/journal.pgph.0002707

**Published:** 2023-12-19

**Authors:** Temesgen Lera, Amene Abebe, Eskinder Wolka, Esayas Aydiko

**Affiliations:** 1 School of Public Health, Wolaita Sodo University, Wolaita, Ethiopia; 2 School of Nursing, Wolaita Sodo University, Wolaita, Ethiopia; Wachemo University, ETHIOPIA

## Abstract

Birth trauma is described as organ or tissue damage caused by physical pressure during birth. Birth trauma ranges from minor problems that go away on their own to significant wounds that might result in infant morbidity and mortality over the long term. It is one of the critical issues that have received the least attention globally. In Ethiopia, evidence regarding the pooled prevalence of birth trauma among neonates is scarce. Therefore, this study estimated the pooled prevalence of birth trauma and associated factors in Ethiopia over the last decade. The Preferred Items for Systematic Reviews and Meta-analysis (PRISMA-2009) guidelines were followed. The PubMed, Scopus, Web of Science, Google Scholar and Google databases were searched. Articles published in the English language in the last decade were included. Data were extracted by a pre-prepared Excel sheet, and analysis was conducted using STATA version 14. Subgroup analysis was also undertaken to evaluate how the prevalence of birth trauma differs across regions of Ethiopia. The pooled prevalence of birth trauma in Ethiopia was 10.57% (95% CI: 3.08, 18.07), with a heterogeneity index (*I*^*2*^) of 92.6% (p < 0.001). Presentation other than vertex AOR 11.94 (95% CI: 6.25–17.62), P = 0.001 and I^2^ = 53%, labor assisted by forceps AOR 6.25 (95% CI: 2.95–10.10), P = 0.002 with I^2^ = 51.8% and labor assisted with vacuum AOR 17.47 (95% CI: 4.25–39.46), P = 0.0001 with I^2^ = 92.9% were factors associated with the pooled prevalence of birth trauma in Ethiopia. The pooled prevalence of birth trauma in Ethiopia is considerable. Non-vertex presentation, use of instrumental delivery and prolonged labor were factors significantly associated with birth trauma. Strengthening neonatal improvement activities (thermal protection, hygienic umbilical cord and skin care, early and exclusive breastfeeding, assessment for signs of serious health problems or need for additional care and preventive treatment), is need.

## Introduction

Birth trauma is described as organ or tissue damage caused by physical pressure during birth. Hypoxia-related injury is disregarded, regardless of whether it can be prevented [1]. According to the International Classification of Diseases 10th revision (ICD-10) and different literature, the common types of birth injuries include birth asphyxia and birth trauma (soft tissue injuries (bruises, petechial, subcutaneous fat necrosis, ulceration, and perforation), extra-cranial hemorrhages (cephalhaematoma, caput succedaneum, subgalial hemorrhage), intracranial hemorrhages, neurological injury (spinal cord injury, facial nerve palsy, brachial plexus injury such as Erb’s palsy and Klumpke’s palsy), and musculoskeletal injury (long bone and clavicular fracture) [2–5].

Birth trauma ranges from minor problems that go away on their own to significant wounds that might result in infant morbidity and mortality over the long term [6–11]. It is a neonatal health issue that receives the least attention globally [12–15]. Worldwide, birth injuries have decreased significantly due to advancements in obstetrical care and prenatal diagnosis [16,17]. However, it is still a significant neonatal concern in low- and middle-income countries [18,19], with the largest rates occurring in Africa and Southeast Asia [20,21].

On average, 10% of the 2.4 million neonates died in 2019 due to birth trauma [22]. The 2018 World Health Organization (WHO) ranking report states that in low- and middle-income nations, birth trauma causes 0.13 to 33 cases per 100,000 population [23]. In Ethiopia, there were 16.25 birth trauma-related deaths for every 100,000 people [23].

Birth trauma is an important contributing factor that raises the burden of ill health and death [21]. Birth trauma also contributes significantly to both short- and long-term malformations and impairments [24].

The prevalence and risk factors for birth trauma differ from country to country, but more crucially, they are affected by access to standardized obstetric care [25]. In many nations, studies have determined the causes of birth trauma. These risk factors can be divided into maternal, fetal, or obstetric complications and lack of poor obstetric care processes, such as lack of antenatal care follow-up, maternal diabetes mellitus, prolonged and obstructed labor, macrosomia, instrumental delivery, malpresentations, maternal age, prematurity, skilled health professionals, low birth weight, and multiple pregnancies [26–28].

Although the Ethiopian Federal Ministry of Health has been performing efforts to lower newborn mortality and increase survival, less than fifty percent of deliveries are attended by trained medical professionals, and a significant number of birth trauma-related deaths are documented [29].

Reducing birth trauma is one of the Sustainable Development Goal 3 objectives [30]. Many previous studies in Ethiopia have attempted to assess the magnitude of birth trauma and birth injury, including birth asphyxia and associated factors [20,31–35]. These studies showed that presentation other than vertex, forceps assist delivery, vacuum assist delivery, fetal distress, standard referral, normal vaginal delivery, birth weight, age, no antenatal attendance and educational status of mother were significantly associated with birth trauma. The findings are inconsistent among studies. There is no study that shows a comprehensive figure of birth trauma and associated factors in Ethiopia. This systematic review and meta-analysis method was chosen to provide strong evidence regarding this issue. Understanding how common birth trauma is and the factors that contribute to it might help create strategies for its early detection and implementation of effective intervention. Therefore, this study aimed to assess the pooled prevalence of birth trauma and associated factors in Ethiopia.

## Methods

### Search strategy

The Preferred Items for Systematic Reviews and Meta-analysis guidelines were followed. The search for published articles was performed using different databases. We systematically searched the PubMed, Scopus, Web of Science, Embase, Google Scholar and Google databases by using the terms “birth trauma”, “birth injury”, “prevalence”, and “magnitude”, and Ethiopia was combined by “and”(S1 Text). Articles published in the English language in the last decade were searched. This systematic review and meta-analysis followed the PRISMA guidelines for literature search strategy, selection of studies, data extraction, and result reporting (S1 Checklist) [36]. To download, organize, review, and site-related articles, EndNote (version X7) reference management software for Windows was used.

### Criteria for article selection

#### Inclusion criteria

Articles published in the English language, articles on the prevalence/magnitude of birth trauma/injury and associated factors among neonates. Articles published in the last decade in Ethiopia were included (from 31 March 2013 to 31 March 2023). Observational studies were included.

#### Exclusion criteria

Case reports, reviews, methodologically poor studies and interventional studies were excluded from this review.

#### The quality assessment of included studies

The quality of each study was assessed using the modified Newcastle‒Ottawa Scale (NOS) for cross-sectional studies [37]. The scale contains eight sections and evaluates the included articles based on selection, comparability, exposure assessment, and outcome. The point scores were as follows: points of 0–5 were considered low quality, 6–7 were considered moderate quality and 8–10 were considered high quality (S1 Table). We included articles with a minimum NOS score of 6. Two authors (TL & AA) assessed the quality of each study based on the NOS. Two authors (EW and EA) assessed titles and abstracts of the identified studies for relevance for review. The full text of relevant articles was retrieved and checked by the primary author.

#### Data extraction and synthesis

Before the actual data extraction began, a pilot extraction of the data using Microsoft Excel was conducted. The first author’s name, publication year, study location, design, sample size, response rate, prevalence, and participants were all included in the data extraction template (S1 Data). Each study was carefully read by two authors (AA and EW), and the prevalence/magnitude of birth trauma/injury and associated factors were identified and extracted to a pre-prepared data extraction Excel sheet. The two authors (AA and EW) separately extracted the data, and any disagreements with the third author (EA) were discussed. After extracting data by Excel, the data were exported to STATA version 14.

#### Definition of terms

**Birth trauma:** Any physical injury to newborns during the entire birth process that can be recognized by clinical physical examination [34].

**Prolonged labor:** Defined as when the combined duration of the first and the second stages of labor are more than 12 h in primipara or 8 h in multipara mothers [34].

**Birth injury:** Injury to newborns that occurs during labor and delivery who have a diagnosis of birth trauma, birth asphyxia or both [34].

#### Data analysis

Data analysis was conducted using STATA version 14. Data exported from Excel were analyzed using STATA version 14. A funnel plot was used to check the publication bias of the studies via personal observation of authors, and a forest plot was used to check the analysis results briefly using CIs. A random effect model was used to estimate the pooled prevalence of birth trauma. To determine whether significant heterogeneity exists, *I*^2^ statistic was used which measures the percentage of variation that is not due to chance. Subgroup analysis between regions of Ethiopia was conducted, and sensitivity analysis of studies was tested.

## Results

### Study selection

**Fig 1:**
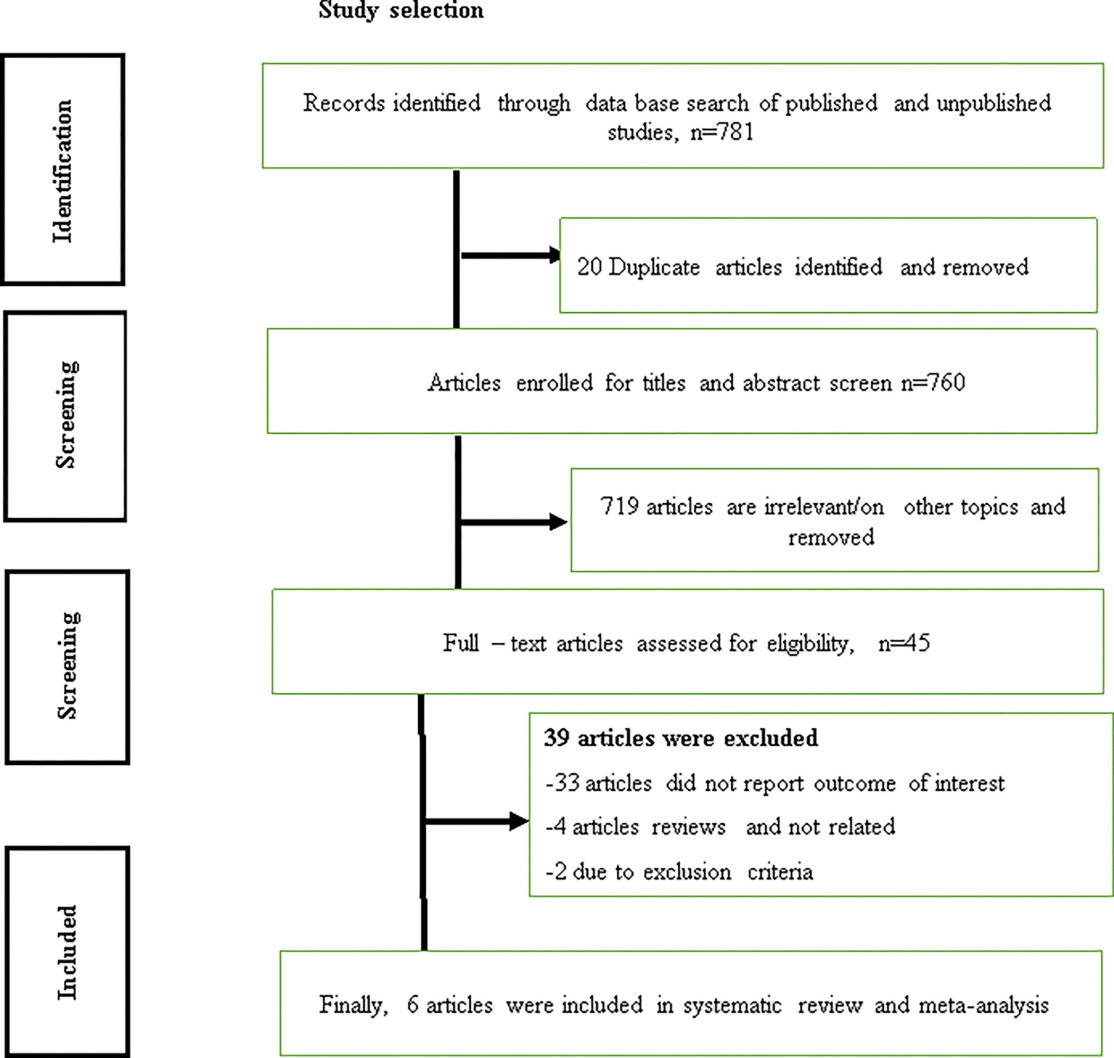
Flow chart of study selection for meta-analysis of birth trauma among neonates in Ethiopia, 2023.

In the beginning, 781 studies were first gathered via database searches. Fifteen duplicates were detected from this and eliminated. Seven hundred nineteen unnecessary studies were eliminated after screening based on their title and abstract. The remaining 45 articles were evaluated for eligibility, and 39 of them were excluded for not reporting the desired outcome, not reporting birth trauma prevalence, or not being primary studies. At the end, 6 articles met the inclusion criteria and were included in the study (Fig 1).

A total of 6 articles with 7085 neonates from Ethiopia were included in this systematic review and meta-analysis. All 6 included studies were cross-sectional in design. The sample size across the studies ranged from 272 [38] to 3814 [35] neonates. Two studies were included from Addis Ababa City and one study from Oromia, Amahara, and the southwestern region and nationwide was obtained. The lowest prevalence of birth trauma/injury (0.472%) was reported in a nationwide study [35], and the highest (16.7%) was reported in the southwestern region [32] (Table 1).

**Table 1:** Characteristics of the included studies in the systematic review and meta-analysis for the prevalence of birth trauma among neonates in Ethiopia, 2023.

No	Author/s name(Reference)	Publication year	Region	Study design	Sample size	Prevalence % 95%CI	NOS
1	Workneh T. et al. [20]	2016	Oromia	Cross-sectional	272	8.1(95%CI: 5.01,12.24)	6
2	Aynalem eta al [31]	2019	Addis Ababa	Cross- sectional	717	12.3 (95% CI:9.84,15.11)	6
3	KetemawuN.et al [32]	2022	South West Region	Cross- sectional	1315	16.7 (95% CI: 14.7, 18.7)	7
4	Gebeyawu B. eta al [33]	2022	Amahara	Cross-sectional	594	13.13 (95%CI:10.30, 16.0)	7
5	Neamin T. et al.[35]	2022	Nationwide study Ethiopia	Cross- sectional	3814	0.472(95% CI: 0.28,0.74)	7
6	Tibebu EA. eta al [34]	2023	Addis Ababa	Cross- sectional	373	12.9 (95% CI:9.49,17.07)	7

### Magnitude of birth trauma among neonates in Ethiopia

**Fig 2:**
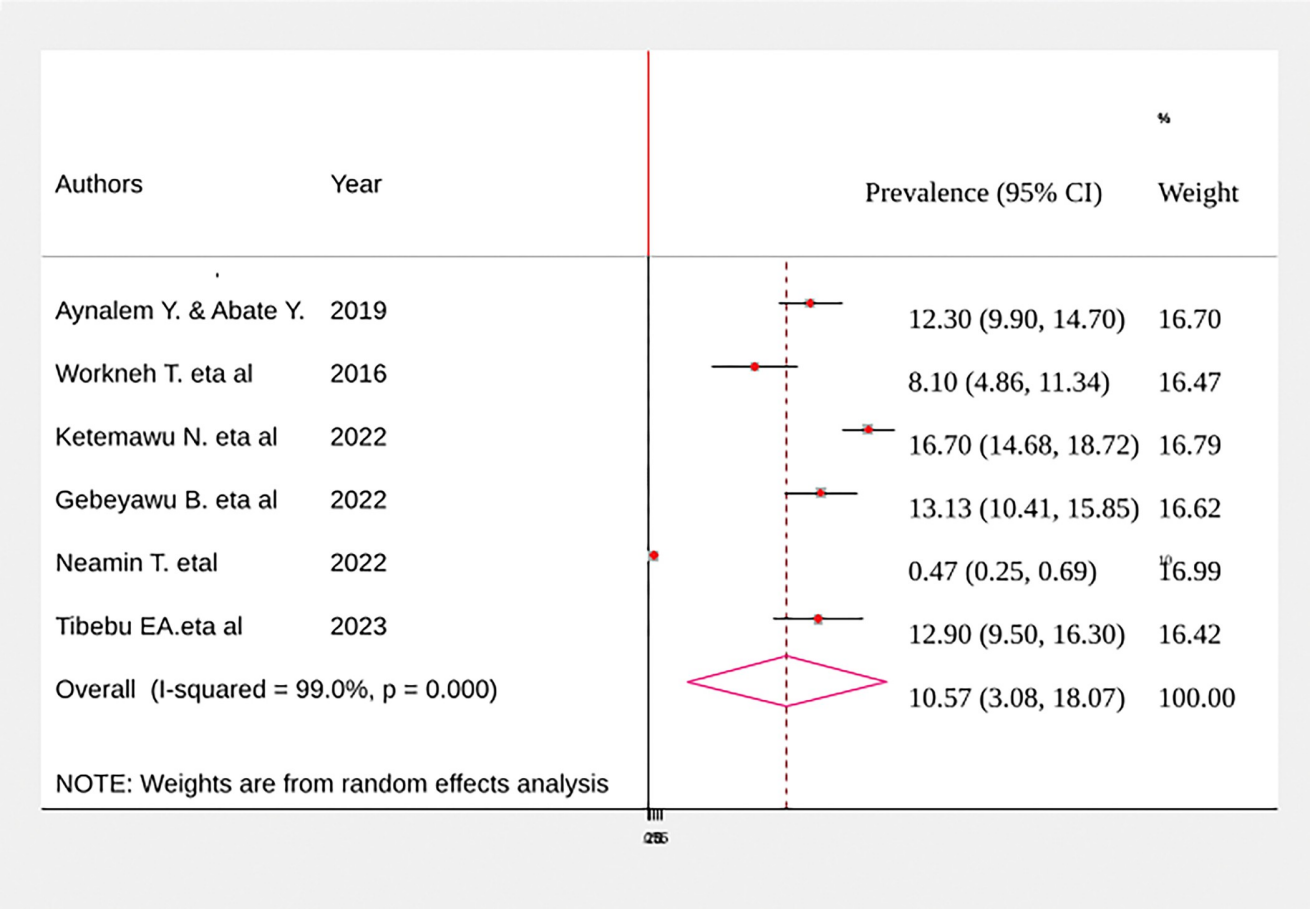


Due to very large reported differences (heterogeneity) among studies, a DerSimonian and Laird random effect model was fitted to determine the pooled effect size. Based on the estimate, the pooled prevalence of birth trauma among neonates was 10.57% (95% CI: 3.08, 18.07), with a heterogeneity index (I^2^) of 92.6% (p < 0.001) (Fig 2).

### Subgroup analysis

**Fig 3:**
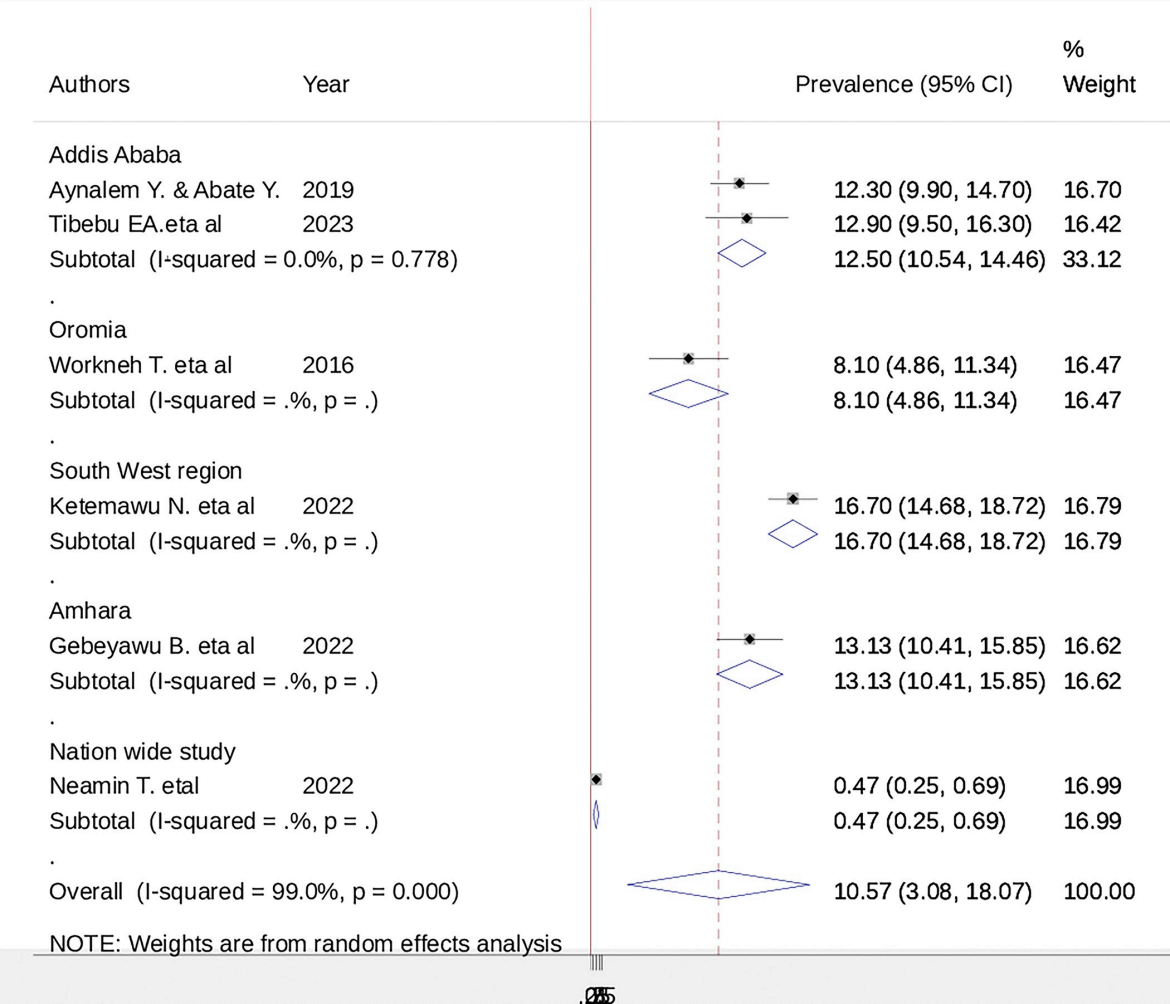


Subgroup analyses revealed a marked variation between regions; the highest prevalence of birth trauma/injury was seen in Addis Ababa (16.7: 95% CI: 14.68, 18.72), and the lowest was seen in a nationwide study conducted in Ethiopia (0.47:95%, 0.25, 0.69) (Fig 3).

### Heterogeneity and publication bias

To determine whether significant heterogeneity exists, *I*^2^ statistic was used which measures the percentage of variation that is not due to chance. The calculated heterogeneity index (I^2^) of 92.6% (p < 0.001). To pinpoint the cause of the reported heterogeneity of this study (I^2^ = 92.6%), we conducted meta-regression employing sample size and publication year as covariates. According to regression, sample size and year of publication, there was no effect of sample size or year of publication on heterogeneity between studies. To adjust and minimize the reported heterogeneity of this study, we performed a subgroup analysis based on the regions in Ethiopia (Table 2).

**Table 2:** Meta-regression analysis of factors affecting between-study heterogeneity.

Heterogeneity source	Coefficients	Std. Err.	P value
Publication year	1	0.729	0.26
Sample size	-2.09	0.0001	1

**Fig 4:**
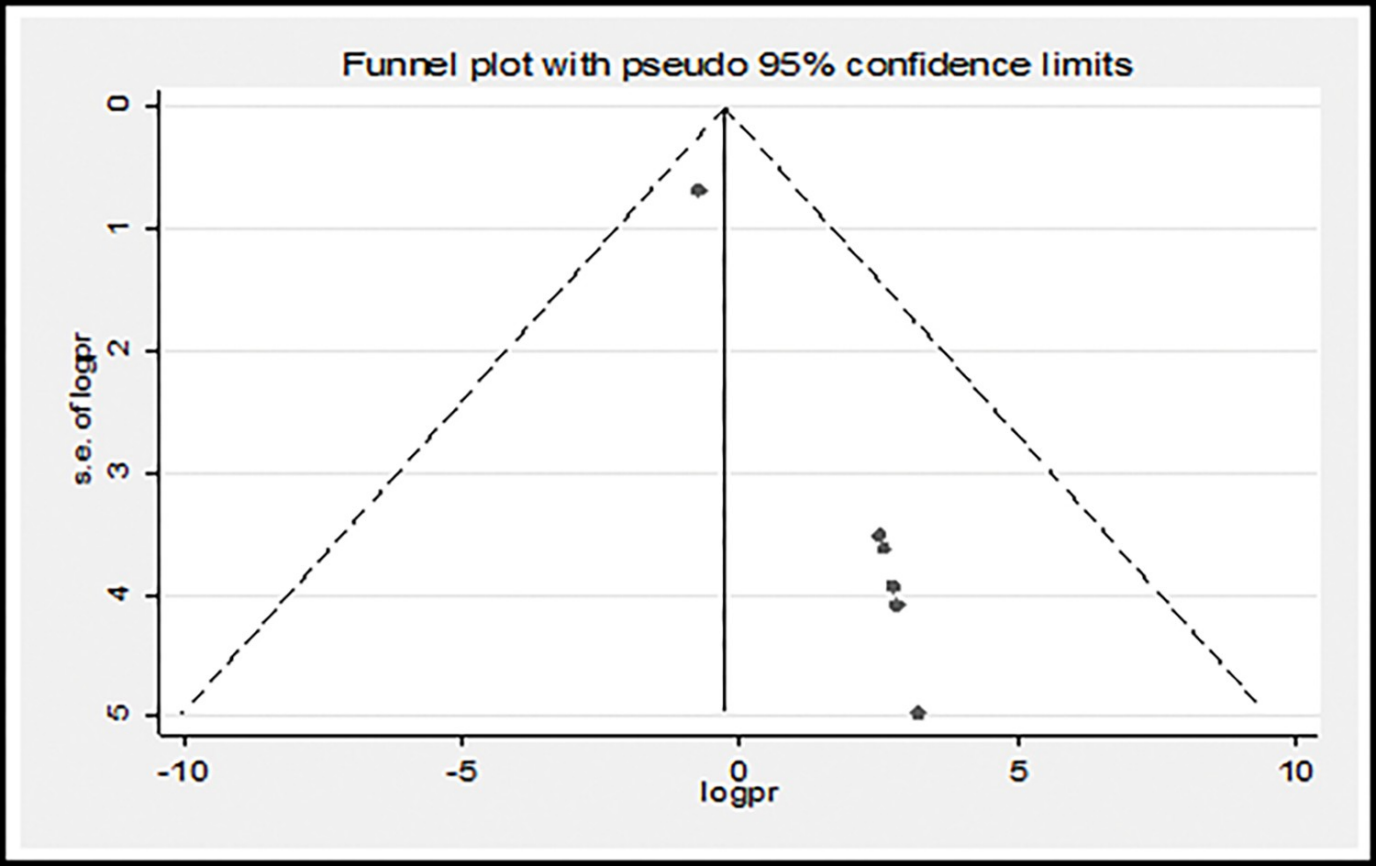


A funnel plot was used to assess publication bias subjectively, and the regression-based Egger’s test was used to assess publication bias objectively at P< 0.05. As seen in (Fig 4), where the funnel plot is asymmetrical since the number of studies on the left and right sides of the plot are different, publication bias has been identified, which is statistically significant, as evidenced by Egger’s test (p < 0.001).

**Fig 5:**
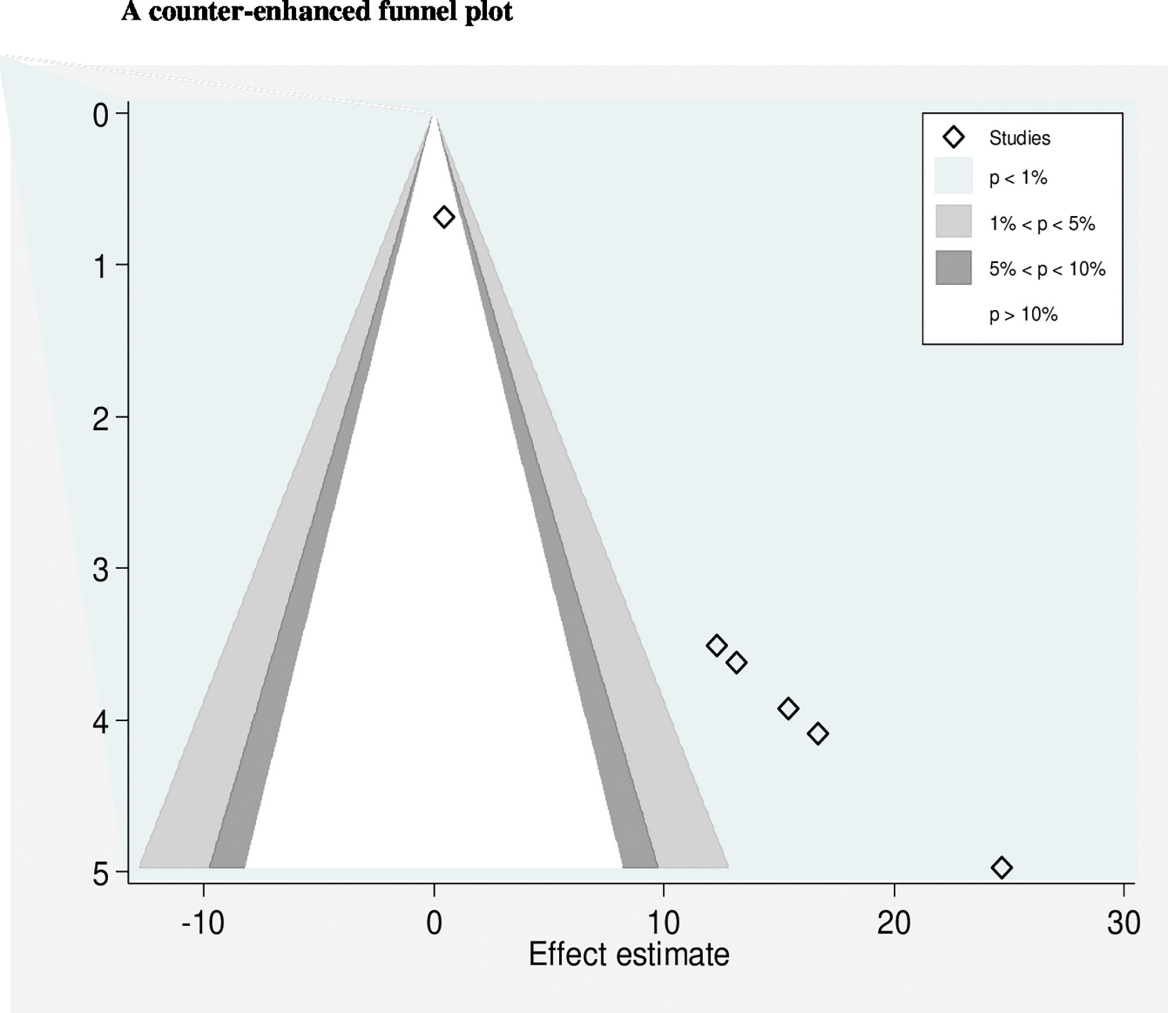
A counter-enhanced funnel plot to explore the causes of publications bias.

Therefore, trim and fill analysis using the random effect model was performed to determine the final effect size. However, a similar effect size was obtained using the model. Furthermore, a counter enhanced funnel plot was used to explore the causes of publication bias Fig 5), which shows that studies appear to be missing in areas of low statistical significance, suggesting that the asymmetry is due to publication bias [20,31–35]. We also executed sensitivity analysis by removing studies step by step to evaluate the effect of a single study on the overall effect estimate. The results indicated that removing a single study did not have a significant influence on pooled prevalence (Table 3).

**Table 3:** Sensitivity analysis of pooled prevalence for each study being removed one at a time.

Study omitted	Publication year	Estimate [95% CI]
Aynalem eta al	2019	2.85(0.8, 10.1)
Workneh T. et al	2016	2.86 (0.81, 10.1)
Ketemawu N. et al	2022	2.87(0.81, 10.1)
Gebeyawu B. et al	2022	2.85(0.805, 10.11)
Neamin T. et al	2022	41(1.58, 1085)
Tibebu EA.et al	2023	2.85 (0.805, 10.11)

### Factors associated with birth trauma in Ethiopia, 2023

**Fig 6:**
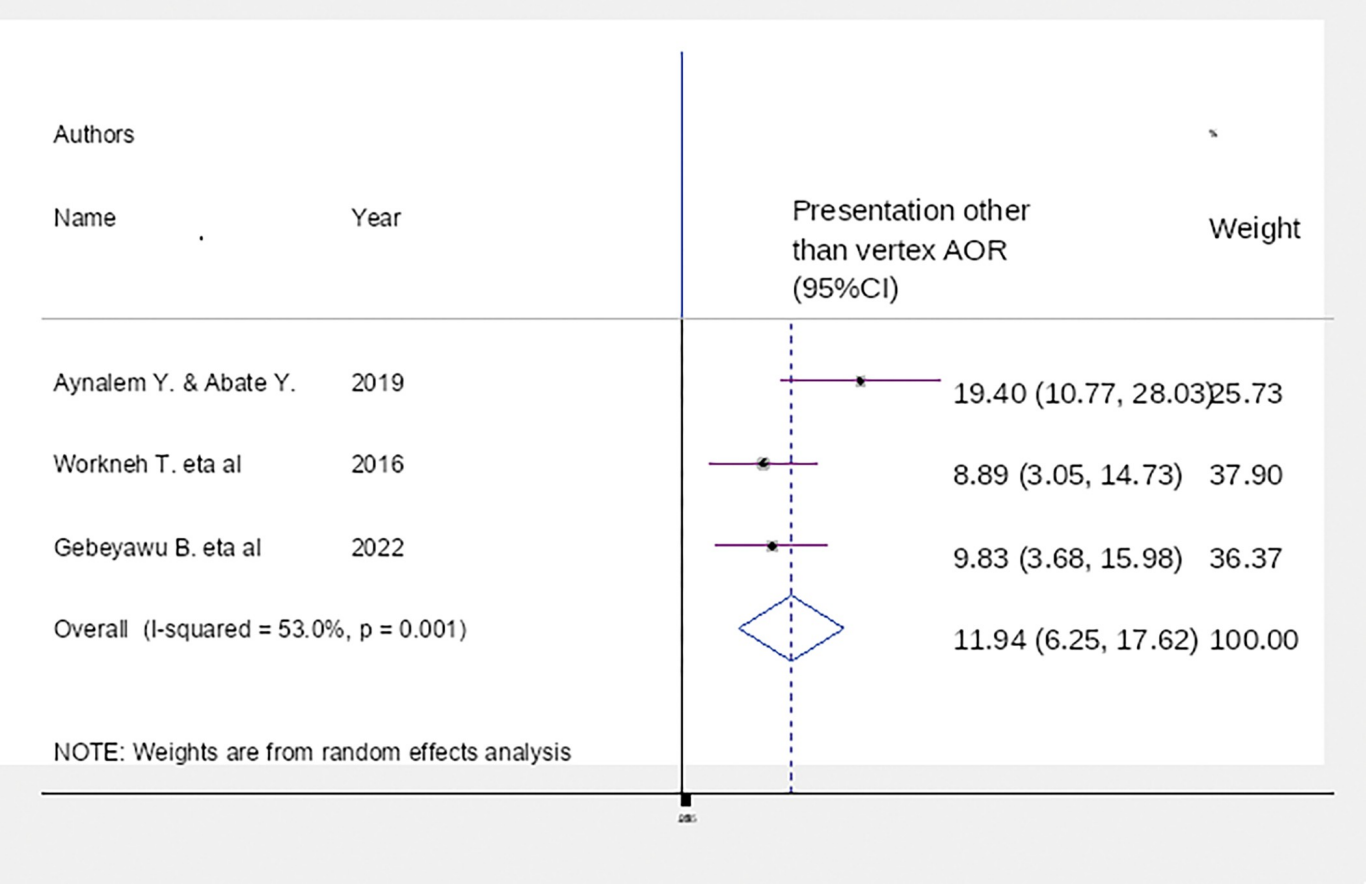


Ten variables (presentation other than vertex, forceps assist delivery, vacuum assist delivery, fetal distress, standard referral, normal vaginal delivery, birth weight, age, no antenatal attendance and educational status of mother) were extracted to identify factors associated with birth trauma occurrence among neonates. Of these, five variables (presentation other than vertex, forceps-assisted delivery, and vacuum-assisted delivery) were found to be significantly associated with the occurrence of birth trauma. Neonates with presentation other than vertex were 11.94 (95% CI: 6.25–17.62), P = 0.001 and I^2^ = 53% more likely to be traumatized than neonates with vertex presentation (Fig 6).

**Fig 7:**
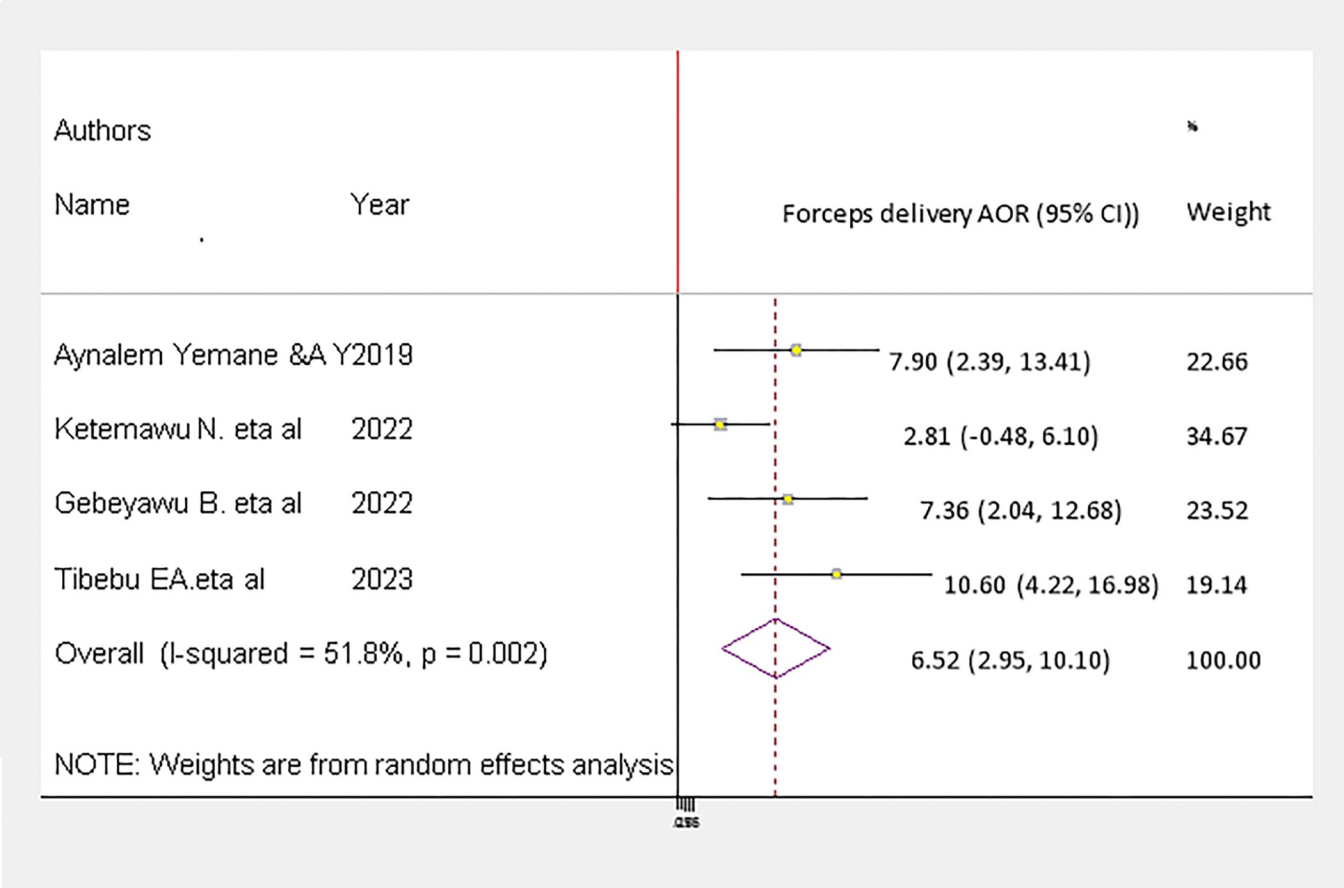


This study revealed that neonates delivered with the assistance of forceps were 6.25 (95% CI: 2.95–10.10), P = 0.002 with I^2^ = 51.8% more likely to develop birth trauma than neonates delivered without forceps assistance (Fig 7).

**Fig 8:**
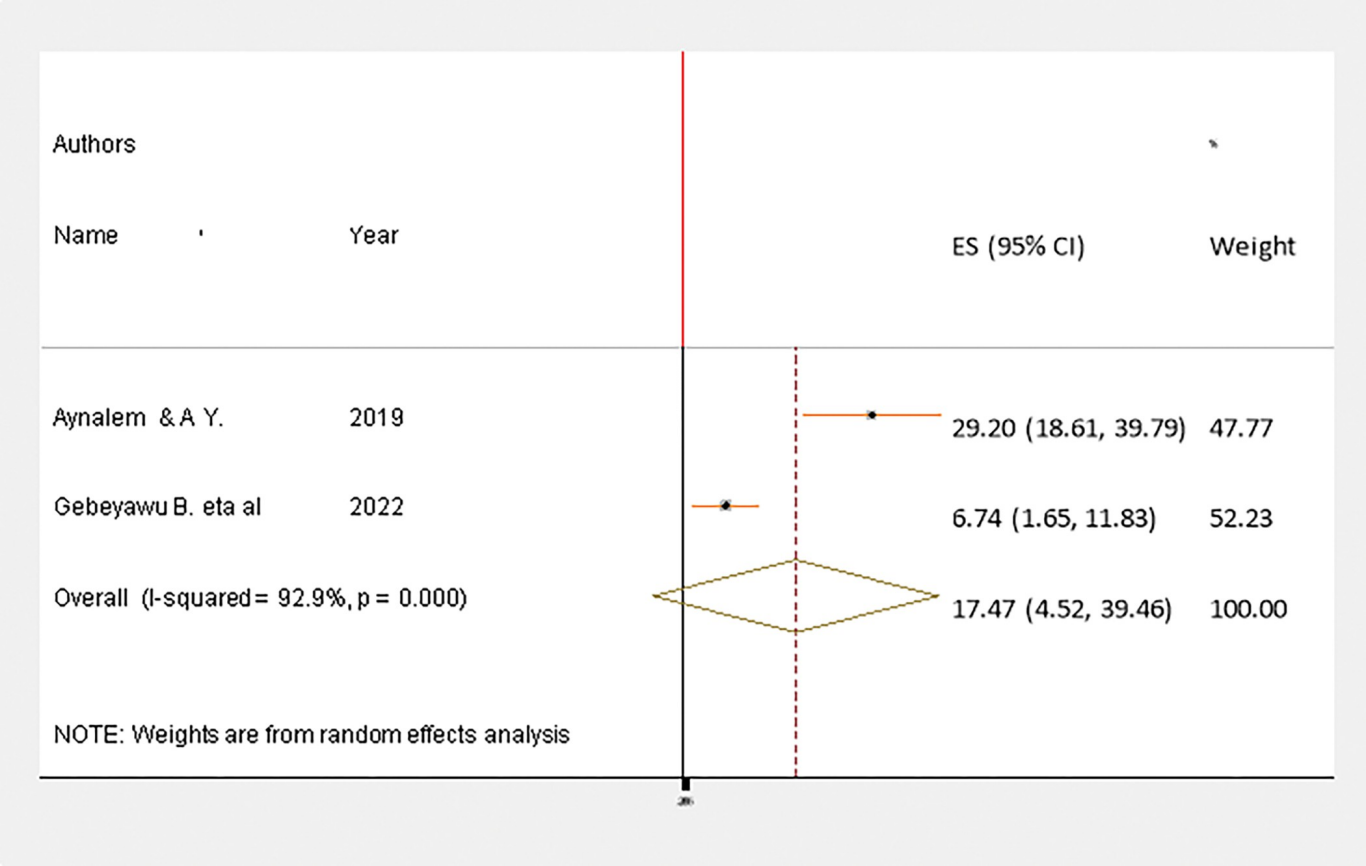


Neonates delivered with the assistance of vacuum were 17.47 (95% CI: 4.25–39.46), P = 0.0001 with I^2^ = 92.9% more likely to develop birth trauma than neonates delivered without vacuum assistance (Fig 8).

## Discussion

This study was conducted to determine the pooled prevalence of birth trauma in Ethiopia. The pooled prevalence of birth trauma among neonates in Ethiopia was 10.57% (95% CI: 3.08, 18.07), with a heterogeneity index (*I*^*2*^) of 92.6% (p < 0.001). This finding is comparable with single studies conducted in Ethiopia in the Amahara region [33] and in Addis Ababa [34]. The findings of the current study are lower than those of studies conducted in Ethiopia in Oromia and the southwestern region [20,32] (15.4% and 16.7%, respectively). The difference may be related to interventions undertaken by different organizations to improve the overall quality of neonatal health.

The current finding is lower than that of a study performed in Romania (16.2%) [38]. This difference might be due to variations in the study time, sample size and study setting. The results of this study are higher than those of the primary study conducted in Kashan, Iran, in which birth trauma among neonates was 2.2% [2], and the systematic review and meta-analysis performed in Iran, in which the pooled prevalence of birth trauma was 2.7% [39]. This difference might be due to the differences in medical technologies and human resources and health facility readiness with trained human resources.

Subgroup analysis based on region showed a variation in the prevalence of birth trauma. This could be because of variations in the study time, sample size and study setting.

In the subgroup analysis, the highest prevalence was observed in Addis Ababa, and the lowest was observed in a nationwide study conducted in Ethiopia by taking samples from different regions. This may be because the number of deliveries attended is high in Addis Ababa compared to regions.

Subgroup analysis based on region showed a variation in the prevalence of birth trauma and significant heterogeneity among the regions. This could be because of variations in the study time, sample size and study setting.

These reviews revealed that neonates who had non-vertex presentation were 11.94 (95% CI: 6.25–17.62), P = 0.001 and I^2^ = 53% times more likely to be traumatized than neonates with vertex presentation. This finding is similar to studies conducted in Jimma [20] and Addis Ababa [34]. This is because non-vertex presentations are prone to prolonged labor and are more likely to be assisted by instrumental deliveries that expose neonates to birth trauma.

The current findings showed that neonates assisted by forceps were 6.25 (95% CI: 2.95–10.10), P = 0.002 with I^2^ = 51.8% more likely to develop birth trauma than neonates delivered without forceps assistance. This finding is comparable with studies conducted in Ethiopia [31–34]. The most plausible explanation was that using forceps on the fetal head could result in extra cranial hemorrhage, intracranial hemorrhage, and soft tissue trauma or laceration, and these complications could result in birth trauma.

This study showed that neonates delivered with the assistance of vacuum were 17.47 (95% CI: 4.25–39.46), P = 0.0001 with I2 = 92.9% more likely to develop birth trauma than neonates delivered without vacuum assistance. This result is in line with studies performed in Ethiopia [31,32]. The likely explanation was that using a vacuum on the fetal head could result in extra cranial hemorrhage, intracranial hemorrhage, and soft tissue trauma or laceration, and these complications could result in birth trauma.

## Limitations of the study

This systematic review and meta-analysis provides up-to-date evidence regarding the prevalence of birth trauma in Ethiopia; however, there are some limitations that need to be considered. First, the protocol for this study was not registered; second, we could not obtain primary studies from some regions of Ethiopia. This study was reported from five regions and one nationwide survey, which might lack representativeness. Despite this limitation, an extensive search was conducted to minimize all possible risks of bias.

## Conclusions

This study revealed that the pooled prevalence of birth trauma in Ethiopia was considerable. Fetal presentations other than vertex, delivery assist by forceps and delivery assist by vacuum were found to be significantly associated with the occurrence of birth trauma. Therefore, strengthening neonatal improvement activities (thermal protection, hygienic umbilical cord and skin care, early and exclusive breastfeeding, assessment for signs of serious health problems or need for additional care and preventive treatment), timely referral from lower health facilities to higher facilities and minimizing instrumental delivery are needed for the prevention of birth trauma.

## Supporting information

S1 Checklist Prisma 2009 checklist. (PDF)

S1 Text Search strategy. (PDF)

S1 Table The quality assessment result of included studies. (PDF)

S1 Data Data extraction template. (XLSX)
