# Supplementary material for: Prevalence and associated factors of birth trauma in Ethiopia: A systematic review and meta-analysis
Source: PLOS Glob Public Health. 2023 Dec 19;3(12):e0002707. doi: 10.1371/journal.pgph.0002707 (PMC10729985; doi:10.1371/journal.pgph.0002707)
Supplement: S1 Text — (PDF) [file pgph.0002707.s002.pdf]

((((birth [Title/Abstract] AND trauma[Title/Abstract]) OR (Birth[Title/Abstract] AND injury[Title/Abstract])) AND Prevalence[Title/Abstract]) OR magnitude[Title]) AND Ethiopia [Title/Abstract].

Terms

Birth Trauma and Birth Injury are used in Google scholar and Google search by adding factors associated with.
