# Supplementary material for: Prevalence and associated factors of birth trauma in Ethiopia: A systematic review and meta-analysis
Source: PLOS Glob Public Health. 2023 Dec 19;3(12):e0002707. doi: 10.1371/journal.pgph.0002707 (PMC10729985; doi:10.1371/journal.pgph.0002707)
Supplement: S1 Table — (PDF) [file pgph.0002707.s003.pdf]

S.№	Article title	Source	Selection				Comparability	Outcome		Total	Quality
			Representativeness of sample	Sample Size	Non-responses	Ascertainment of exposure (Disease)		Assessment of outcome	Statistical test		
1.	Tesfaye W, Workneh N. Girma E: birth injury and associated factors in jimma university specialized Hospital, Southwest Ethiopia. Ethiopian Journal of Pediatrics and Child Health 2016;12:20–31.	Workneh T. et al.	0	1	1	1	1	1	1	6	Moderate
2.	Birth trauma among neonates admitted to neonatal unit in a tertiary hospital in Addis Ababa	Aynalem Y.& Abate Y.et al.	0	1	1	1	1	1	1	6	Moderate
3.	Prevalence and associated factors of birth injury among neonates admitted at neonatal intensive care unit (NICU) in governmental hospitals of Southwest Ethiopian people regional state, Ethiopia: A multicenteric cross-sectional study	Ketemawu N.eta l	1	1	1	1	1	1	1	7	High
4.	Magnitude of birth trauma and its associated factors in South Wollo public hospitals, northeast Ethiopia, August	Gebeyawu B. eta al	1	1	1	1	1	1	1	7	High

	2021: Institutional-Based Cross Sectional Study										
5.	Cause and risk factors of early neonatal death in Ethiopia.	Neamin T. etal	1	1	1	1	1	1	1	7	High
6.	Prevalence of birth injuries and associated factors among newborns delivered in public hospitals Addis Ababa, Ethiopia, 2021. Crossectional study	Tibebu EA. eta al	1	1	1	1	1	1	1	7	High
